# (Salen)Mn(III) Catalyzed Asymmetric Epoxidation Reactions by Hydrogen Peroxide in Water: A Green Protocol

**DOI:** 10.3390/ijms17071112

**Published:** 2016-07-12

**Authors:** Francesco Paolo Ballistreri, Chiara M. A. Gangemi, Andrea Pappalardo, Gaetano A. Tomaselli, Rosa Maria Toscano, Giuseppe Trusso Sfrazzetto

**Affiliations:** 1 Department of Chemical Sciences, University of Catania, Viale Andrea Doria 6, 95125 Catania, Italy; fballistreri@unict.it (F.P.B.); gangemichiara@unict.it (C.M.A.G.); andrea.pappalardo@unict.it (A.P.); 2 I.N.S.T.M. UdR of Catania, Viale A. Doria 6, 95125 Catania, Italy

**Keywords:** epoxidation, enantioselectivity, jacobsen catalyst, hydrogen peroxide, water

## Abstract

Enantioselective epoxidation reactions of some chosen reactive alkenes by a chiral Mn(III) salen catalyst were performed in H_2_O employing H_2_O_2_ as oxidant and diethyltetradecylamine *N*-oxide (AOE-14) as surfactant. This procedure represents an environmentally benign protocol which leads to e.e. values ranging from good to excellent (up to 95%).

## 1. Introduction

Nowadays, there is a growing requirement to design green synthetic protocols to reduce or to eliminate the use and generation of hazardous substances [1,2,3]. Metal-Salen complexes are a wide class of organometallic compounds that have been found applications in several fields, such as catalysis [4,5], imaging [6], solar cells [7,8] and sensing [9,10,11,12,13]. In particular, asymmetric epoxidation of unfunctionalized alkenes catalyzed by chiral (salen)Mn(III) complexes has proved to be a reaction of industrial and pharmacological importance, leading to the desired products via the synthetically versatile epoxy function [14,15,16,17]. Since the employment of water as reaction medium is particularly appealing for achieving mild, cheap and environmentally benign reaction conditions, in a previous work, we have developed an aqueous reaction medium, based on aqueous surfactant solutions, for the enantioselective epoxidation of 1,2-dihydronaphthalene and various *cis*-β-alkyl styrenes using the Jacobsen chiral (salen)Mn(III) as catalyst, but bleach as oxidant [18]. Nevertheless, the oxidation processes are going to abandon the use of hazardous oxidants (e.g., bleach) preferring greener ones, such as hydrogen peroxide [2,19,20].

Hydrogen peroxide represents the oxidant of choice because it has many attractive properties, such as its high active oxygen content (47%), its reduction product that is water and the availability of an inexpensive oxidant aqueous solution (30%) that is easy to handle [21]. By way of the contrast, the main problem in using aqueous hydrogen peroxide in the (salen)Mn(III) catalyzed epoxidations is related to the hydrogen peroxide decomposition (homolitic cleavage of O–O bond) catalyzed by the salen itself [22] and to the concomitant deactivation of the catalyst by hydrogen peroxide [22,23,24,25].

In the literature, the examples of enantioselective epoxidations utilizing manganese salen complexes in association with hydrogen peroxide as the primary oxidant are not very numerous [2,26,27,28,29,30]. Most of them employ manganese salen catalysts, bearing a covalently bound internal pyridine or an *N*-methyl-imidazole ligand, an ureido group, or tertiary amine *N*-oxides as cocatalysts [24,25]. In fact, the heterolytic O–O bond cleavage yielding the oxene, (salen)Mn(V)=O, which is the real oxidant species [31,32], is favoured by these bases [33,34,35,36]. However, in all these studies, the main solvent is not water but an organic solvent (e.g., CH_2_Cl_2_/CH_3_OH 1/1, CH_2_Cl_2_/DMF 1/3 and CH_2_Cl_2_) to prevent potential solubility problems [24,37,38]. An environmentally friendly protocol for salen catalyzed enantioselective epoxidation reactions would envisage hydrogen peroxide as oxidant and water as a reaction medium. In such a case, the decomposition of hydrogen peroxide, the degradation/deactivation of the catalyst, and the lack of solubility of organic substrates in water are the disadvantages to face.

These drawbacks might be minimized under reaction conditions where the alkene epoxidation reaction is quite fast to compete with oxidant decomposition (reactive alkenes) and, at the same time, the catalyst is stabilized by suitable coligands. In addition, hydrogen peroxide, in excess with respect to the stoichiometric ratio, can counterbalance its decomposition.

**Figure 1:**
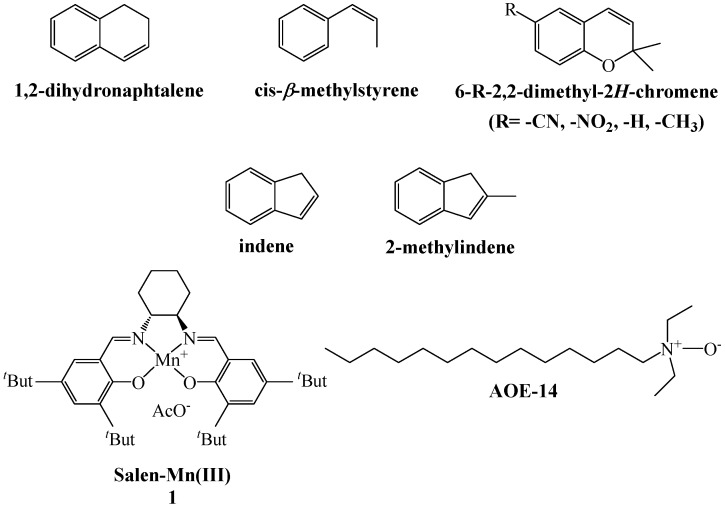
Alkenes, Mn(III) catalyst and surfactant with relative acronyms used in this work.

Herein, we report a green protocol for the (salen)Mn(III) catalytic enantioselective epoxidation of non-functionalized alkenes (see Figure 1), which utilizes aqueous H_2_O_2_ as terminal oxygen donor and water as a reaction medium under homogeneous conditions.

## 2. Results

To overcome the solubility problems of alkenes in water, we selected diethyltetradecylamine *N*-oxide (AOE-14) as surfactant because, as we already reported [18], it solubilizes organic reagents in water, but it also is able to work as cocatalyst, binding the metal of the chiral catalyst (Equation (1)) and improving the catalyst stability, the reaction rate, as well as the enantioselectivity:

L + Cat ⇔ L-Cat(1)

With L = AOE-14, Cat = (salen)Mn(III), where the catalyst L-Cat bearing the coligand L is more stable and more efficient than the catalyst without coligand. As far as L-Cat identity is concerned, we have determined by LC-MS a molecular ion with *m*/*z* = 885, which is the expected mass of L-Cat (see Figure S1).

As a matter of fact, Jacobsen et al. [39] have reported that amine *N*-oxide additives have a dramatic impact on the outcome of the epoxidation reaction, affecting the rate, yield, *cis*/*trans* ratio and enantioselectivity, suggesting that the additive stabilizes the highly reactive oxene O=Mn(salen) species by ligation after the initial oxidation [31,32]. In addition, X-ray crystal structures demonstrated that *N*-oxides additives function as axial ligands. Furthermore, Senanayake et al. [40,41] reported a study to explore the reactivity of Jacobsen catalyst in the presence of various *N*-oxides, such as P_3_NO (i.e., 4-(3-Phenylpropyl)pyridine *N*-oxide). They noted that P_3_NO binds the catalyst (C=N stretching at 1613 cm^−1^ is shifted to 1623 cm^−1^), reduces the catalyst decomposition, exhibits rate acceleration depending on the concentration ratio additive/catalyst, increases e.e. values.

Therefore, the epoxidation reactions of some reactive standard alkenes (Figure 1), using H_2_O_2_ as oxidant and catalyzed by Jacobsen (salen)Mn(III), were carried on at 25 °C in water in the presence of AOE-14, as both surfactant and cocatalyst. Preliminary experiments were performed to set up optimal reaction conditions (Table 1).

Entries 1–5 indicate that the enantioselective epoxidation of 6-CN-2,2-dimethylchromene is quite fast but, after 20 min, the reaction stops since the same value of conversion both at 20 min and after 5 h is observed. The addition of a second aliquot of catalyst induces the restarting of the epoxidation reaction (entry 6) and, 10 min after this further addition, a high conversion value (81%) is detected. On the other hand, the e.e. values are quite good and not depending on the conversion degree as expected. The presence of probable deactivation processes of catalyst, competing with the epoxidation reaction, might be responsible of this observed behavior.

It is reported that both the demetalation and the ligand degradation cause the instability of the salen complex, particularly when the catalytic reaction requires the presence of strong acids or of oxidizing/reducing reagents [23]. The salen ligand can undergo attack by oxidant at the imine site or at other sites suffering degradation [23]. Demetalation leads to the metal-free salen ligand, that is very prone to undergo hydrolysis to the corresponding salicylaldehyde and diamine.

Check experiments seem to support the salen complex degradation by H_2_O_2_ in water. In fact, a solution of 0.002 M catalyst in water, in the presence of 0.1 M AOE-14, after 24 h, does not show the formation of degradation products (i.e., 3,5-di-*tert*-butylsalicylaldehyde). However, degradation products appear only after addition of H_2_O_2_ to the mixture. ^1^H NMR spectra confirm the presence of free salen ligand and trace of 3,5-di-*tert*-butylsalicylaldehyde (see Figure S2). Under these experimental conditions, the catalyst degradation process is quite clear after 4 hours. However, the doubling of the AOE-14 concentration (0.2 M) makes the epoxidation reaction rate able to efficiently compete with the deactivation reaction (Table 1, entries 7–9).

Therefore all reactions were performed under these experimental conditions using [alkene] = 0.05 M, [H_2_O_2_] = 0.4 M, [catalyst] = 0.002 M and [AOE-14] = 0.2 M, and pertinent results are reported in Table 2 and Table 3. For all studied chromenes, e.e. values are very good (>80% up to 95%) as well as conversion values, except for nitro derivative, which shows a lower conversion value of 61%. On the other hand, it can be observed that the conversion values are lower also for *cis*-β-methylstyrene, 1,2-dihydronaphthalene, indene and 2-methylindene, probably because the epoxidation rates for these alkenes, under the adopted experimental conditions, are lower than those of chromenes and therefore catalyst deactivation processes are competing more efficiently with the epoxidation reactions.

In order to verify whether it is possible to obtain a reactivity increase for these alkenes toward the oxidant and, therefore, to compete and to overcome the parallel degradation reactions, we performed new experiments with *cis*-β-methylstyrene, 1,2-dihydronaphthalene, indene and 2-methylindene increasing the concentrations of the catalyst, as well as of the coligand. The pertinent results are reported in Table 3.

**Figure 2:**
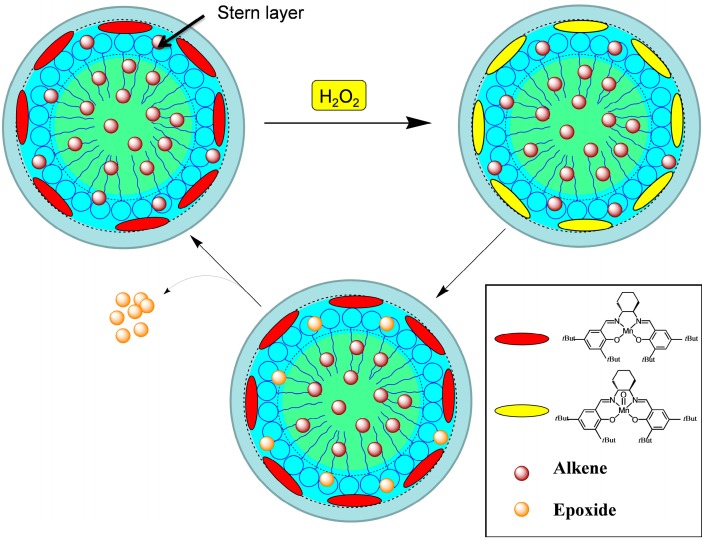
Schematic representation of the catalytic cycle.

It can be seen that, in the case of *cis*-β-methylstyrene, employing a catalyst double concentration and increasing the coligand concentration up to 4 times (from 0.2 M to 0.8 M), an increase of conversion from 50% to 73% is observed (Table 3, entries 14–16). Likewise, in the case of 1,2-dihydronaphthalene (Table 3, entries 17–19), a doubling of catalyst concentration and an increase of coligand concentration up to 4 times leads to a 100% of conversion. Additionally, in the case of indene (Table 3, entries 20–21), a double concentration of the catalyst and an increase of coligand concentration up to 3 times leads to a conversion value two times higher (66%). In the case of 2-methylindene (entries 22–24), again, the increase of the catalyst concentration, as well as of the coligand, leads to a higher conversion value of 87%. Therefore, it is interesting to observe that conversion values are correlated to the [AOE-14]/[catalyst] ratio, that increase on increasing this ratio and also for the less reactive alkenes are from good to excellent. This behavior can be rationalized considering that the surfactant, which works also as coligand, at concentration values larger than 1.1 × 10^−4^ M (AOE-14 c.m.c., (see Materials and Methods) forms micelles, whose polar heads, i.e., the *N*-oxides groups, bind molecules of catalyst. Therefore, our system might be envisaged as constituted by nanoreactors [42] in which the active catalyst is located, through binding of the *N*-oxides polar heads to the metal site, in the micelles. Epoxidation reaction would occurs within these nanoreactors in the Stern layer catalyzed by the salen molecules bound to the micelles themselves (Figure 2) and the shielding effect of hydrophilic micelle shell would give a more efficient and selective catalysis (e.g., local reactant concentration higher than bulky concentration) [42].

The larger the [AOE-14]/[catalyst] ratio the higher the number of micelles, acting as reaction reactors, bearing the bound catalyst molecules. A relationship between the conversion values and the [AOE-14]/[catalyst] ratios can be observed in Table 3 for some of the alkenes undergoing epoxidation reactions. When most catalyst molecules are bound to the micelles, a saturation effect trend can be observed (see Figure S3).

## 3. Materials and Methods

### 3.1. General

NMR experiments were carried out at 27 °C on a Varian Unity Inova 500 MHz spectrometer (^1^H NMR at 499.88 MHz, ^13^C NMR at 125.7 MHz) equipped with pulse field gradient module (*Z* axis) and a tunable 5 mm Varian inverse detection probe (ID-PFG, Agilent, Santa Clara, CA, USA). The chemical shifts (ppm) were referenced to TMS (^1^H, 0.0 ppm) or CDCl_3_ (^13^C, 77.0 ppm). ESI mass spectra were acquired on an ES-MS Thermo-Finnigan spectrometer (Thermo Fisher Scientific, Waltham, MA, USA) equipped with an ion trap analyzer.

Enantiomeric excesses were determined by GC analysis using a Perkin Elmer Capillary (Perkin Elmer, Waltham, MA, USA) and HPLC (Agilent, Santa Clara, CA, USA) analysis using a Varian Pro-Star-RI Detector, equipped with dual cell refractometer using a column packed with an appropriate optical active material, as described below. TLC analysis was performed on silica gel 60 F_254_-aluminium sheets (0.25 mm, Merck, Darmstadt, Germany).

The absolute configuration of the obtained epoxides were determined by measuring the optical rotation with a polarimeter. Absolute configurations were assigned by comparison of the measured [α]_D_^2^° values with those reported in the literature [43]. (Salen)Mn(III) was synthesized following the procedure reported in the literature [44,45]. Critical micelle concentration of AOE-14 was determined by surface tension measurements (private communication by Raimondo Germani, Department of Chemistry, University of Perugia, Perugia, Italy).

### 3.2. Preparation of Alkenes

6-CN-2,2-dimethylchromene, 6-NO_2_-2,2-dimethylchromene, 6-H-2,2-dimethylchromene, 6-CH_3_-2,2-dimethylchromene were synthesized as reported in literature [46]. *cis*-β-methylstyrene is obtained from the corresponding commercial alkyne by hydrogenation with the Lindlar catalyst in cyclohexane according to the following procedure [47].

### 3.3. Enantioselective Epoxidation in Surfactant Solutions

In a typical run, alkene was added to a stirred solution of surfactant and catalyst in distilled water (2 mL); after the complete solubilization of the alkene, H_2_O_2_ was added to the mixture and the reaction was kept in a round-bottom flask at 25 °C in a thermostatic bath. After a certain reaction time, the aqueous solution was extracted with 1 mL of CH_2_Cl_2_. Combined organic extracts were dried over anhydrous MgSO_4_, reduced to a small volume, and analyzed by GC or HPLC as described above. Isolation of 6-CN-2,2-dimethylchromene epoxide, as representative example, was carried out by the following procedure: after a certain reaction time, the aqueous solution was extracted with CH_2_Cl_2_, combined organic extracts were dried over anhydrous MgSO_4_, and the epoxide was isolated by chromatography on silica gel (*N*-hexane/EtOAc 9/1). The identity of the compound was confirmed by ^1^H NMR and ESI-MS (Thermo Fisher Scientific, Waltham, MA, USA).

### 3.4. Product Analysis

Gas chromatographic analyses of the reaction mixtures were carried out on a gas chromatograph equipped with a flame ionization detector and program capability. The e.e., yields and conversions values were determined employing the chiral column DMePeBETACDX (25 m × 0.25 mm ID × 0.25 μm film; MEGA, Legnano, Italy) for 1,2-dihydronaphthalene, indene and 2-methylindene (isotherm 150 °C), the chiral column DMeTButiSililBETA-086 (25 m × 0.25 mm ID × 0.25 μm film; MEGA) for *cis*-β-methyl styrene (column conditions: 50 °C (0 min) to 120 °C (1 min) at 2 °C/min). The injector and detector temperatures were maintained at 250 °C for all columns, *N*-dodecane was used as an internal standard throughout. For chromene epoxides, e.e. and conversion values were determined by HPLC analysis using a chiral stationary phase column (Lux 5μ cellulose-3, PHENOMENEX; *N*-hexane/*i*PrOH 9:1) and by ^1^H NMR spectroscopic analysis using chiral shift reagent (+)Eu(hfc)_3_.

## 4. Conclusions

This enantioselective epoxidation protocol of alkenes by hydrogen peroxide in water in the presence of AOE-14, in the dual role of surfactant and cocatalyst, gives good to excellent results in terms of conversion values and enantiomeric selectivities. The protocol seems suitable for a large variety of alkenes of different reactivity because it is possible the tuning of the reaction conditions by an appropriate choice of the [AOE-14]/[catalyst] ratio. In addition, allowing the use of water as reaction medium and hydrogen peroxide as oxidant, it represents an environmentally and ecologically benign procedure which contributes to enrich the library of asymmetric epoxidation reactions green chemistry.

## Figures and Tables

**Table 1:** Enantioselective epoxidation of 6-CN-2,2-dimethylchromene with H_2_O_2_ catalyzed by (salen)Mn(III) in H_2_O in the presence of AOE-14 at 25 °C. ^a^

Entry	[AOE-14] (M)	Time (min)	Conv. ^b^ (%)	e.e. ^b^ (%)
1	0.1	1	7	84
2	0.1	3	12	81
3	0.1	10	35	80
4	0.1	20	58	82
5	0.1	300	55	83
6 ^c^	0.1	10 ^d^	81	84
7	0.2	10	60	83
8	0.2	20	81	83
9	0.2	300	81	82

^a^ In all experiments [alkene] = 0.05 M, [H_2_O_2_] = 0.4 M, [catalyst] = 2.0 × 10^−3^ M (4%); ^b^ Determined by HPLC analysis using a chiral stationary phase column; ^c^ After 5 h a further amount of catalyst (2.3 × 10^−3^ M) was added; ^d^ 10 min after the addition of the second aliquot of catalyst.

**Table 2:** Enantioselective epoxidation of alkenes with H_2_O_2_ catalyzed by (salen)Mn(III) in H_2_O in the presence of AOE-14 as surfactant at 25 °C. ^a^

Entry	Alkene	Conv. ^d^ (%)	e.e. ^b^ (%)	Conf. ^c^
10	6-CN-2,2-dimethylchromene	81 ^e^	83 ^b,d^	3R,4R
11	6-NO_2_-2,2-dimethylchromene	61	86	3R,4R
12	6-H-2,2-dimethylchromene	87	95	3R,4R
13	6-CH_3_-2,2-dimethylchromene	78	80	3R,4R

^a^ In all experiments: [alkene] = 0.05 M, [catalyst]= 2 × 10^−3^ M, [H_2_O_2_] = 0.4 M, [AOE-14] = 0.2 M, reaction time = 30 min; ^b^ Determined by ^1^H NMR using chiral shift reagent (+)Eu(hfc)_3_; ^c^ Determined by measuring the optical rotation; ^d^ Determined by GC on chiral column (see Materials and Methods); ^e^ Isolated yield 76%.

**Table 3:** Enantioselective epoxidation of alkenes with H_2_O_2_ catalysed by (salen)Mn(III) in H_2_O in the presence of AEO-14 as surfactant at 25 °C. ^a^

Entry	Alkene	[Cat.] (10^−3^ M)	[AOE-14] (M)	Conv. ^b^ (%)	e.e. ^b^ (%)	Ratio ^d^
14	*cis*-β-methylstyrene	2	0.2	50	80 ^c^	100
15	*cis*-β-methylstyrene	4	0.6	70	80 ^c^	150
16	*cis*-β-methylstyrene	4	0.8	73	80 ^c^	200
17	1,2-dihydronaphthalene	2	0.2	44	54	100
18	1,2-dihydronaphthalene	4	0.6	92	54	150
19	1,2-dihydronaphthalene	4	0.8	100 ^e^	54	200
20	indene	2	0.2	30	80	100
21	indene	4	0.6	66	80	150
22	2-methylindene	1	0.2	25	90	50
23	2-methylindene	2	0.6	51	91	100
24	2-methylindene	4	0.8	87	91	200

^a^ In all experiments: [alkene] = 0.05 M, [H_2_O_2_] = 0.4 M, reaction time = 30 min; ^b^ Determined by GC on chiral column (see Materials and Methods); In all cases, configurations are (1R, 2S); ^c^ Enantiomeric excess (e.e.) value is referred to the to the major *cis* epoxide (*cis*/*trans* = 4); ^d^ [AOE-14]/[Catalyst]; ^e^ isolated yield 89%.

## Supplementary Materials

Supplementary materials can be found at http://www.mdpi.com/1422-0067/17/7/1112/s1.

## References

[1] Srour H., Le Maux P., Chevance S., Simouneaux G. (2013). Metal-catalyzed asymmetric sulfoxidation, epoxidation and hydroxylation by hydrogen peroxide. Coord. Chem. Rev..

[2] De Faveri G., Ilyashenko G., Watkinson M. (2011). Recent advances in catalytic asymmetric epoxidation using the environmentally benign oxidant hydrogen peroxide and its derivatives. Chem. Soc. Rev..

[3] Lane B.S., Burgess K. (2003). Metal-catalyzed epoxidations of alkenes with hydrogen peroxide. Chem. Rev..

[4] McGarrigle E.M., Gilheany D.G. (2005). Chromium- and manganese-salen promoted epoxidation of alkenes. Chem. Rev..

[5] Shi Q.-P., Shi Z.-H., Li N.-G., Tang Y.-P., Li W., Tang H., Zhang W., Shen M.-Z., Duan J.-A. (2013). Asymmetric epoxidation of olefins with homogeneous chiral (salen) manganese(III) complexes. Curr. Org. Chem..

[6] Jing J., Chen J.J., Hai Y., Zhan J., Xu P., Zhang J.L. (2012). Rational design of ZnSalen as a single and two photon activatable fluorophore in living cells. Chem. Sci..

[7] Yang S., Kou H., Wang H., Cheng K., Wang J. (2010). Efficient electrolyte of *N,N*’-*bis*(salicylidene) ethylenediamine zinc(II) iodide in dye-sensitized solar cells. New J. Chem..

[8] Shoji R., Ikenomoto S., Sunaga N., Sugiyama M., Akitsu T. (2016). Absorption wavelength extension for dye-sensitized solar cells by varying the substituents of chiral salen Cu(II) complexes. J. Appl. Sol. Chem. Mod..

[9] D’Urso A., Tudisco C., Ballistreri F., Condorelli G.G., Randazzo R., Tomaselli G.A., Toscano R.M., Trusso Sfrazzetto G., Pappalardo A. (2014). Enantioselective extraction mediated by a chiral cavitand–salen covalently assembled on a porous silicon surface. Chem. Commun..

[10] Tudisco C., Trusso Sfrazzetto G., Pappalardo A., Motta A., Tomaselli G.A., Fragalà I.L., Ballistreri F.P., Condorelli G.G. (2011). Covalent functionalization of silicon surfaces with a cavitand–modified salen. Eur. J. Inorg. Chem..

[11] Ballistreri F.P., Pappalardo A., Tomaselli G.A., Toscano R.M., Sfrazzetto G.T. (2010). Heteroditopic chiral uranyl–salen receptor for molecular recognition of amino acid ammonium Salts. Eur. J. Org. Chem..

[12] Amato M.E., Ballistreri F.P., D’Agata S., Pappalardo A., Tomaselli G.A., Toscano R.M., Sfrazzetto G.A. (2011). Enantioselective molecular recognition of chiral organic ammonium ions and amino acids using cavitand–salen-based receptors. Eur. J. Org. Chem..

[13] Pappalardo A., Amato M.E., Ballistreri F.P., Tomaselli G.A., Toscano R.M., Trusso Sfrazzetto G. (2012). Pair of diastereomeric uranyl salen cavitands displaying opposite enantiodiscrimination of α-amino acid ammonium Salts. J. Org. Chem..

[14] Jacobsen E.N., Ojima I. (1993). Catalytic Asymmetric Synthesis.

[15] Katsuki T., Ojima I. (2004). Catalytic Asymmetric Synthesis.

[16] Sfrazzetto G.T., Millesi S., Pappalardo A., Toscano R.M., Ballistreri F.P., Tomaselli G.A., Gulino A. (2015). Olefin epoxidation by a (salen)Mn(III) catalyst covalently grafted on glass beads. Cat. Sci. Technol..

[17] Amato M.E., Ballistreri F.P., Pappalardo A., Tomaselli G.A., Toscano R.M., Williams D.J. (2005). Novel chiral (salen)MnIII complexes containing a calix[4]arene unit as catalysts for enantioselective epoxidation reactions of (Z)-aryl alkenes. Eur. J. Org. Chem..

[18] Ballistreri F.P., Brinchi L., Germani R., Savelli G., Tomaselli G.A., Toscano R.M. (2008). Enantioselective epoxidations of alkenes catalyzed by (salen)Mn(III) in aqueous surfactant systems. Tetrahedron.

[19] Brink G.-J., Arends I.W.C.E., Sheldon R.A. (2004). The Baeyer-Villiger reaction: New developments toward greener procedures. Chem. Rev..

[20] Wang C., Yamamoto H. (2015). Asymmetric Epoxidation Using Hydrogen Peroxide as Oxidant. Chem. Asian J..

[21] Colladon M., Scarso A., Sgarbossa P., Michelin R.A., Strukul G. (2007). Regioselectivity and diasteroselectivity in Pt(II)-mediated “green” catalytic epoxidation of terminal alkenes with hydrogen peroxide: Mechanistic insight into a peculiar substrate selectivity. J. Am. Chem. Soc..

[22] Salem I.A., El-Sheikh M.Y., Zaki A.B. (1995). Kinetics and mechanism of the decomposition of hydrogen peroxide catalyzed by Mn(II)-bis-salicylaldimine complexes. Monatsh. Chem..

[23] Baleizao C., Garcia H. (2006). Chiral salen complexes: An overview to recoverable and reusable homogeneous and heterogeneous catalysts. Chem. Rev..

[24] Pietikainen P. (1998). Convenient asymmetric (salen)Mn(III)-catalyzed epoxidation of unfunctionalized alkenes with hydrogen peroxide using carboxylate salt cocatalysts. Tetrahedron.

[25] Watanabe Y., Namba A., Umezawa N., Kawahata M., Yamaguchi K., Higuchi T. (2006). Enhanced catalase-like activity of manganese salen complexes in water: Effect of a three-dimensionally fixed auxiliary. Chem. Commun..

[26] Grigoropoulou G., Clark J.H., Elings J.A. (2003). Recent developments on the epoxidation of alkenes using hydrogen peroxide as an oxidant. Green Chem..

[27] Irie R., Hosoya N., Katsuki T. (1994). Enantioselective epoxidation of chromene derivatives using hydrogen peroxide as a terminal oxidant. Synlett.

[28] Schwenkreis T., Berkessel A. (1993). A biomimetic catalyst for the asymmetric epoxidation of unfunctionalized olefins with hydrogen peroxide. Tetrahedron Lett..

[29] Kureshy R.I., Khan N.H., Abdi S.H.R., Singh S., Ahmed I., Shukla Ram S., Jasra R.V. (2003). Chiral Mn(III) salen complex-catalyzed enantioselective epoxidation of nonfunctionalized alkenes using urea-H_2_O_2_ adduct as oxidant. J. Catal..

[30] Matsumoto K., Oguma T., Katsuki T. (2009). Highly enantioselective epoxidation of styrenes catalyzed by proline-derived C_1_-symmetric titanium(salan) complexes. Angew. Chem. Int. Ed..

[31] Feichtinger D., Plattner D.A. (1997). Direct proof for O: MnV(salen) complexes. Angew. Chem. Int. Ed..

[32] La Paglia Fragola V., Lupo F., Pappalardo A., Sfrazzetto G.T., Toscano R.M., Ballistreri F.P., Tomaselli G.A., Gulino A. (2012). A surface-confined O: MnV(salen) oxene catalyst and high turnover values in asymmetric epoxidation of unfunctionalized olefins. J. Mater. Chem..

[33] Battioni P., Renaud J.P., Bartoli J.F., Reina-Artiles M., Fort M., Mansuy D.J. (1988). Monooxygenase-like oxidation of hydrocarbons by hydrogen peroxide catalyzed by manganese porphyrins and imidazole: Selection of the best catalytic system and nature of the active oxygen species. J. Am. Chem. Soc..

[34] Anelli P.L., Banfi S., Montanari F., Quilici S. (1989). Synergistic effect of lipophilic carboxylic acids and heterocyclic axial ligands in alkene epoxidation by hydrogen peroxide catalyzed by manganese(III) tetraarylporphyrins. J. Chem. Soc. Chem. Commun..

[35] Banfi S., Maiocchi A., Montanari F., Quici S. (1990). Hydrogen peroxide epoxidation of olefins catalyzed by manganese(III) tetraarylporphyrins. Synergistic effect of carboxylic acids and of lipophilic heterocyclic axial ligands. Gazz. Chim Ital..

[36] Gonsalves A.M.D.A., Johnstone R.A.W., Pereira M.M., Shaw J. (1991). Metal-assisted reactions. Part 21: Epoxidation of alkenes catalyzed by manganese-porphyrins: The effects of various oxidatively-stable ligands and bases. J. Chem. Soc. Perkin Trans..

[37] Shitama H., Katsuki T. (2006). Asymmetric epoxidation using aqueous hydrogen peroxide as oxidant: Bio-inspired construction of pentacoordinated Mn-salen complexes and their catalysis. Tetrahedron Lett..

[38] Liu S.-Y., Nocera D.G. (2006). A simple and versatile method for alkene epoxidation using aqueous hydrogen peroxide and manganese salophen catalysts. Tetrahedron Lett..

[39] Finney N.S., Pospil P.J., Chang S., Palucki M., Konsler R.G., Hansen K.B., Jacobsen E.N. (1997). On the viability of oxametallacyclic intermediates in the (salen)Mn-catalyzed asymmetric epoxidation. Angew. Chem. Int. Ed..

[40] Senanayake C.H., Smith G.B., Ryan K.M., Fredenburgh L.E., Liu J., Roberts F.E., Hughes D.L., Larsen R.D., Verhoeven T.R., Reider P.J. (1996). The role of 4-(3-phenylpropyl)pyridine *N*-oxide (P_3_NO) in the manganese-salen-catalyzed asymmetric epoxidation of indene. Tetrahedron Lett..

[41] Hughes D.L., Smith G.B., Liu J., Dezeny G. C., Senanayake C.K., Larsen R.D., Verhoeven T.R., Reider P.J. (1997). Mechanistic study of the jacobsen asymmetric epoxidation of indene. J. Org. Chem..

[42] Lu J., Liang L., Weck M. (2016). Micelle-based nanoreactors containing Ru-porphyrin for the epoxidation of terminal olefins in water. J. Mol. Catal. A: Chem..

[43] Scheurer A., Mosset P., Spiegel M., Saalfrank R.W. (1999). Reverse asymmetric catalytic epoxidation of unfunctionalized alkenes. Tetrahedron.

[44] Patti A., Pedotti S., Ballistreri F.P., Sfrazzetto G.T. (2009). Synthesis and characterization of some chiral metal-salen complexes bearing a ferrocenophane substituent. Molecules.

[45] Lombardo G.M., Thompson A.L., Ballistreri F.P., Pappalardo A., Sfrazzetto G.T., Tomaselli G.A., Toscano R.M., Punzo F. (2012). An integrated X-ray and molecular dynamics study of uranyl-salen structures and properties. Dalton Trans..

[46] Bergmann R., Gericke R. (1990). Synthesis and antihypertensive activity of 4-(1,2-dihydro-2-oxo-1-pyridyl)-2*H*-1-benzopyrans and related compounds, new potassium channel activators *J*. Med. Chem..

[47] Foltz C.M., Witkop B.J. (1957). Stereochemistry of the 1-phenyl-1,2-propanediols and of α-isoephedrine. J. Am. Chem. Soc..

