# Supplementary material for: (Salen)Mn(III) Catalyzed Asymmetric Epoxidation Reactions by Hydrogen Peroxide in Water: A Green Protocol
Source: Int J Mol Sci. 2016 Jul 12;17(7):1112. doi: 10.3390/ijms17071112 (PMC4964487; doi:10.3390/ijms17071112)
Supplement: Supplementary file 1 [file ijms-17-01112-s001.pdf]

Supplementary Materials: (Salen)Mn(III) Catalyzed Asymmetric Epoxidation Reactions by Hydrogen Peroxide in Water: A Green Protocol

Francesco Paolo Ballistreri, Chiara M. A. Gangemi, Andrea Pappalardo, Gaetano A. Tomaselli, Rosa Maria Toscano and Giuseppe Trusso Sfrazzetto

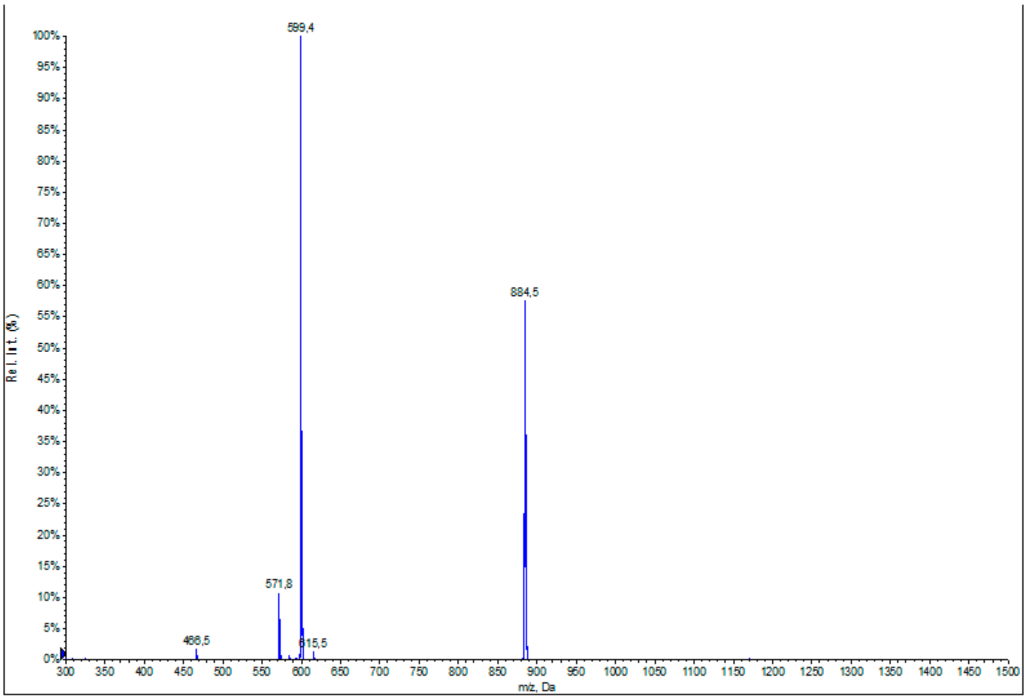

Figure S1. Mass spectrum of complex L-Cat (peak at *m*/*z* 599.4 is relative to the catalyst).

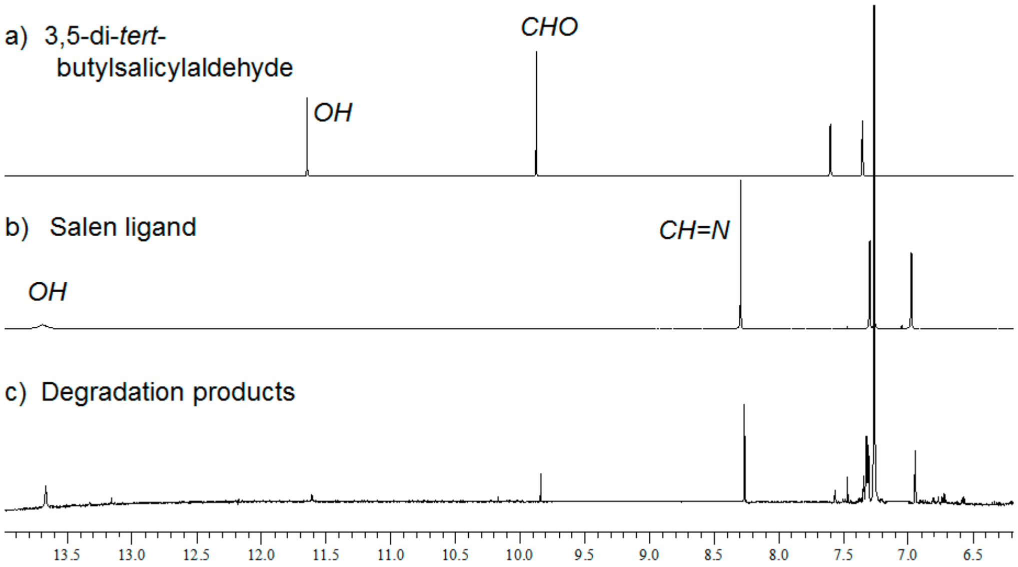

Figure S2. Selected regions of ^1H NMR spectra in $CDCl_3$ of (**a**) 3,5-di-tert-butylsalicylaldehyde; (**b**) salen ligand; (**c**) sample of a solution of (salen)Mn(III)/H_2O_2/AOE-14 after extraction with CH_2Cl_2 (after 4 h) where the signal at 9.8 ppm relative to the CHO proton of 3,5-di-tert-butylsalicyaldehyde and the signals at 8.3 and 13.57 ppm, respectively, relative to the imine CH=N and to the phenolic OH of the free ligand can be observed.

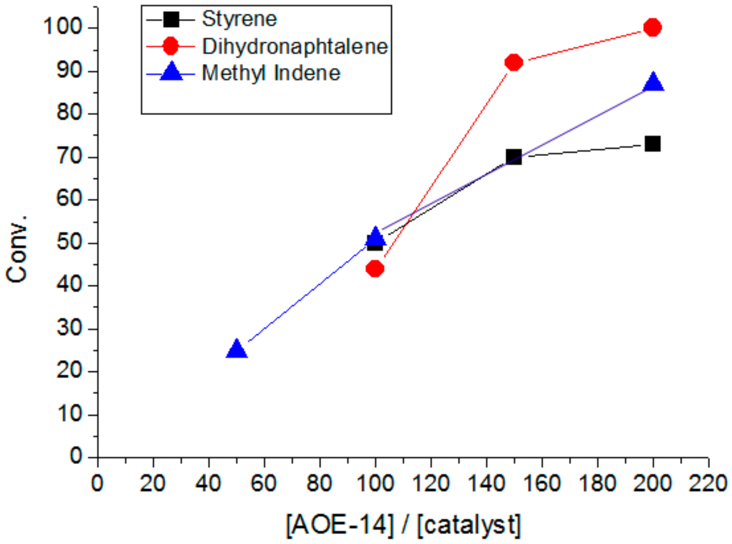

Figure S3. Relationships between the conversion values and the [AOE-14]/[catalyst] ratios.
